# Attitudes of dental students in governmental versus private universities in Egypt towards acquiring a career in dental public health: a cross-sectional study

**DOI:** 10.1186/s12909-026-09280-x

**Published:** 2026-05-04

**Authors:** Ayman El-Naggar, Dina Hamdy, Amira Badran

**Affiliations:** 1 Department of Pediatric Dentistry and Dental Public Health, Faculty of Dentistry, Ain Shams University, Cairo, Egypt; 2 Faculty of Dentistry, Galala University, Al Galala, Egypt

**Keywords:** Dental public health, Career choice, Dental students, Attitudes, Egypt, Governmental and private

## Abstract

**Background:**

Dental Public Health (DPH) plays a critical role in promoting oral health and addressing community needs, yet remains an unpopular career choice among dental graduates. Understanding students’ attitudes toward DPH is essential for strengthening the dental workforce in prevention-oriented fields, particularly in developing countries such as Egypt. The purpose of the study is to assess the attitudes of dental students in governmental and private universities in Egypt toward pursuing a career in DPH, and to examine how demographic characteristics, academic preferences, and opinions about the DPH subject relate to these attitudes.

**Methods:**

A cross-sectional survey was conducted among final-year dental students and interns from six Egyptian dental schools (three governmental, three private). A validated, adapted paper-based questionnaire was administered. Attitudes toward DPH were measured using a five-point Likert scale and categorized as positive, neutral, or negative. Chi-square tests were used to analyze associations between variables.

**Results:**

A total of 393 students participated (response rate: 93.8%). Nearly half (49.9%) showed a positive attitude toward DPH. However, only 6.4% considered pursuing a career in the specialty. A few students (6.4%) favored DPH as an undergraduate subject, and 3.6% preferred it for postgraduate specialization. University type showed no significant association with attitude (*P* = 0.41), nor did gender or area of residence. However, regression analysis demonstrated that public university students were 1.7 times more likely to pursue a career in the specialty. About 51% of the students showed interest in serving the public and working in public service. Significant differences in future career plans were observed between students in governmental and private universities (*P* = 0.03).

**Conclusion:**

Egyptian dental students generally exhibit positive attitudes toward DPH, yet few intend to pursue it as a career. Attitudes were not influenced by university type, gender, or area of residence, but were strongly shaped by students’ opinions of the subject of DPH in the undergraduate curriculum. Strengthening early exposure, enhancing practical components, and promoting the importance of DPH are strategies that have the potential to improve interest in this specialty.

**Supplementary Information:**

The online version contains supplementary material available at 10.1186/s12909-026-09280-x.

## Contributions to the literature

- The study has direct relevance to public health policy, dental workforce planning, and curricular reform in Egypt.
- It identifies key gaps, implications for dental education, and possible areas for reform that could improve students’ engagement with DPH as a subject and as a potential career.
- It provides actionable evidence to support calls for increased early practical exposure, field-based learning, and integration of public health competencies into the national dental curriculum.
- It sheds light on patterns and factors that may be influencing the distribution of the dental workforce in Egypt.

## Introduction

Oral health is a vital component of general health and well-being, yet many populations suffer from preventable dental diseases due to inadequate public health infrastructure, inequitable access, and misalignment between workforce distribution and population needs [1]. Dental Public Health (DPH) is the cornerstone of any dental health care delivery system, as it is the branch of dentistry concerned with the promotion of oral health, aiming at the prevention of oral diseases, [2] and with raising awareness about the social aspects of the dental profession and its responsibility towards the community [3]. It is a unique branch of dentistry that focuses on dental and oral health issues in communities and populations rather than treating patients and dealing with them individually [4].

Egypt’s dental education system has evolved from one dominated by governmental faculties to a mixed ecosystem where private universities have become increasingly influential. Now, more than 40 dental schools are offering the Bachelor of Dental Surgery (BDS) degree, divided among governmental and private universities, which are more than enough to provide a sufficient number of graduates to supply the country with a strong workforce in all branches of dentistry [5]. Governmental dental schools are state-funded with larger student cohorts, forcing budgetary constraints that can limit their access to cutting-edge technology, while private schools that charge substantially higher tuition fees often possess greater financial flexibility to invest in modern and innovative teaching models [6–8]. These institutional and educational differences, along with behavioral differences between the students in both institutes [7]. might impact the students’ career pathways.

However, some dental departments are able to attract dental students and interns in large numbers. Studies have shown that dental students are more likely to choose a clinical specialty such as conservative dentistry, endodontics, orthodontics, oral and maxillofacial surgery, pedodontics, and periodontics, while oral medicine, diagnosis and radiology, oral pathology, and DPH specialties were not popular for postgraduate studies among dental students [9–13]. Unfortunately, it is usually at the expense of other, less popular fields such as DPH [14].

Specialty choice among dental students is driven by a multifactorial interplay of intrinsic and extrinsic motivations, personal and financial influences, academic experiences, and societal and market demands, as proven by several studies [14–19].

Despite its critical role in improving oral health equity, DPH is often underrepresented among dental students’ career preferences [20]. Hesitancy of students to prioritize DPH for postgraduation studies is a cause for concern [21]. Studies have shown that limited exposure during undergraduate training, low financial incentives compared to clinical specialties, and a lack of awareness of DPH career opportunities contribute to this trend [2, 5, 14, 22–24]. However, graduates pursuing a career in DPH can work in various sectors, such as governmental and policy-making roles, academic and research careers, international and non-governmental organizations (NGOs), health service management and administration, community program leadership, and consultancy and advocacy.

To ensure the oral health care system functions sustainably and maintains high-quality dental care, it is essential that every branch of dentistry be adequately staffed [25]. In Egypt, as in many developing countries, strengthening DPH career pathways through structured mentorship, early curricular exposure, and integration of public health principles into clinical training could help address workforce gaps [5, 26].

Dental schools play a key role in influencing attitudes toward the Public Health sector and can influence career choices through their curriculum design while emphasizing the importance of serving the community and promoting professional responsibility towards society [27]. Therefore, it is essential to investigate how dental students in Egypt, across governmental and private institutions, view acquiring a career in DPH. Understanding these attitudes is critical for informing workforce planning, aligning dental curricula with public health needs, and ensuring that oral health services are equitably distributed and responsive to the needs of the community.

Raising the research question “Is there a difference in attitude of dental students in governmental vs. private dental schools in Egypt towards choosing a career in DPH?”, this study aimed to assess the attitude of dental students in Egyptian faculties of dentistry towards DPH as a career choice, focusing on the differences in these attitudes between governmental and private dental schools. It also explores the demographic characteristics of the students, their preferences, and their opinions regarding the subject of DPH. The null hypothesis can be stated as - H_01_: There is no difference in attitude regarding choosing a career in DPH between dental students in governmental universities and those in private universities in Egypt.

## Methods

The proposal for this study was revised by the Ethical Committee of the Faculty of Dentistry, Ain Shams University, which granted it the ethical approval number (FDASU-Rec IM012408) on January 25th of 2024. Participants were informed that submitting the questionnaire is considered consent to their participation and the use of their answers in the study, strictly for research purposes. This study did not include any clinical or experimental procedures; consequently, no harm was expected to the participants other than the time spent responding to the questionnaire. Participants were informed of the purpose of the study and of the confidentiality of their identities and responses.

A cross-sectional study was conducted, implementing a paper-based questionnaire. The study’s design and reporting adhered to the Strengthening the Reporting of Observational Studies in Epidemiology (STROBE) guidelines for cross-sectional studies [28]. The questionnaire was adapted from a previous study by Naidu et al. [29] making some structural modifications while maintaining the original intent of the instrument. A version of the original questionnaire is provided as Supplementary File 1. Sections of the pre-tested survey and certain items were changed from two sections to three sections in our study, in addition to rephrasing certain items to be clearer. Few choices were deemed relevant and were added to the responses; irrelevant items were omitted, such as the item asking about the name, and certain items were necessarily added, including the item asking about the type of university, to meet the aim of our study, and another asking about the most interesting field/s of DPH. The survey was then distributed among 20 students for face validity and to confirm the clarity and comprehensibility of the questionnaire items, with an additional comment section at the end of the survey. The insightful comments of the 20 students prompted the researcher to further edit the questionnaire. Based on these comments, certain items in the main section inquiring about the attitude were then merged or omitted for being repetitive. These brought the item count for the main section to 13 items instead of 16 for the purpose of making the survey briefer and more efficient.

Content validity of the questionnaire was then assessed by a panel of six experts. Both item-level content validity index (I-CVI) and scale-level content validity index, average calculation method (S-CVI/Ave = 0.892) met acceptable values.

The reliability analysis of the 13-item attitude scale demonstrated satisfactory internal consistency, as indicated by a Cronbach’s alpha coefficient of 0.829, which exceeds the commonly accepted threshold of 0.70, confirming that the scale possesses good reliability. The “Cronbach’s alpha if item deleted” values ranged from 0.807 to 0.833, which were all close to the overall alpha value and did not show substantial improvement upon deletion of any individual item. This finding indicates that none of the items negatively affected the internal consistency of the scale and that all statements contributed meaningfully to the overall measurement of attitude.

The final version of the questionnaire consisted of 26 items divided into three sections. Section (1) explores general demographic characteristics of the students: gender, area of residence, type of university, and year of study. Section (2) inquires about their preferences regarding the subjects they are studying as undergraduate students, the department they plan on choosing for their postgraduate studies, and their opinion of the subject of DPH in their curriculum. Section (3) is the main section exploring the attitudes of dental students towards DPH as a future career.

Sample size calculation was based on the prevalence of positive attitude (66.0%) towards DPH retrieved from previous research [20] Using Epi Info version 7.2.4.0 to calculate sample size based on expected prevalence, which reaches 66% with 95% CL (confidence level) with an acceptable margin of error = 5, the sample size was 345 students. This number was increased by 15% to compensate for invalid responses that would be excluded from the study. This will bring the total sample size to 397 students. Divided by six faculties, the survey was handed to 66 students in each faculty. 

Six faculties of dentistry were selected for the study, taking into consideration that they are all geographically distributed across different regions of Egypt. One governmental and one private dental school were chosen from each of Greater Cairo, the Nile Delta, and Upper Egypt, using convenient cluster selection, followed by simple random sampling. For eligibility of participation, only final (5th ) year dental students and dental interns who have already studied DPH as part of their undergraduate curriculum were eligible for the study. The distinction between final year students and interns should be noted, as Bachelor of Dentistry BDS (BDS) programs in Egypt are five-year long, followed by a 6th year of internship [5].

Data collection commenced in February of the year 2025 at the beginning of the second semester and ended in June of the same year. Printed questionnaires were handed to the participants in each faculty at the beginning or the end of normal clinical classes in the faculty clinics or lectures in the lecture halls. The time needed to answer the whole survey was 10–15 min.

The participants’ responses to the attitude items of the questionnaire were graded on a five-point Likert scale system as follows: *Strongly agree (4)*,* Agree (3)*,* Undecided (2)*,* Disagree (1)*,* and Strongly disagree (0).* This scale brings the minimum and maximum scores of the 13-item attitudes section to 0 and 52, respectively. The maximum score was divided by three to categorize the level of attitude into three tiers, which are: *negative (scores range: 0–17)*, *neutral (scores range: 18–34)*, and *positive (scores range: 35–52)* attitudes. This method of categorization followed previous comparable studies [23, 30] However, the score ranges differ from these studies, since they implement a 16-item attitudes section with a maximum score of 64.

Analysis of data was done using the SPSS program version 27. Chi-square test was used to compare categorical variables between different groups and a P-value < 0.05 was considered statistically significant. Fisher’s Exact test was used when the expected cell count is less than 5 in > 25% of cells.

A multivariable logistic regression analysis was performed to identify the independent predictors of students’ intention to pursue a career in DPH. The model included demographic and academic variables (age, gender, residence, university type, and year of study) along with the categorized attitude scores (negative, neutral, and positive). The ‘Enter’ method was utilized to determine the adjusted odds ratios (aOR) and their 95% confidence intervals (CI). Reference groups were defined as females for gender, governmental institutions for university type, and low attitude for the attitude scale.

## Results

A total of 393 responses of the 419 handed-in surveys were submitted. Twenty-six responses were excluded for being incomplete or blank. Among the 393 students, 211 were females (53.7%), and 182 were males (46.3%). Urban residents constituted 70.2% of the participants, while fewer came from rural settings (29.8%). Students were almost evenly split between governmental universities (49.4%) and private universities (50.6%). The majority of the students were in their final (5th ) year of study (75.1%), while 24.9% were interns.

**Fig. 1:**
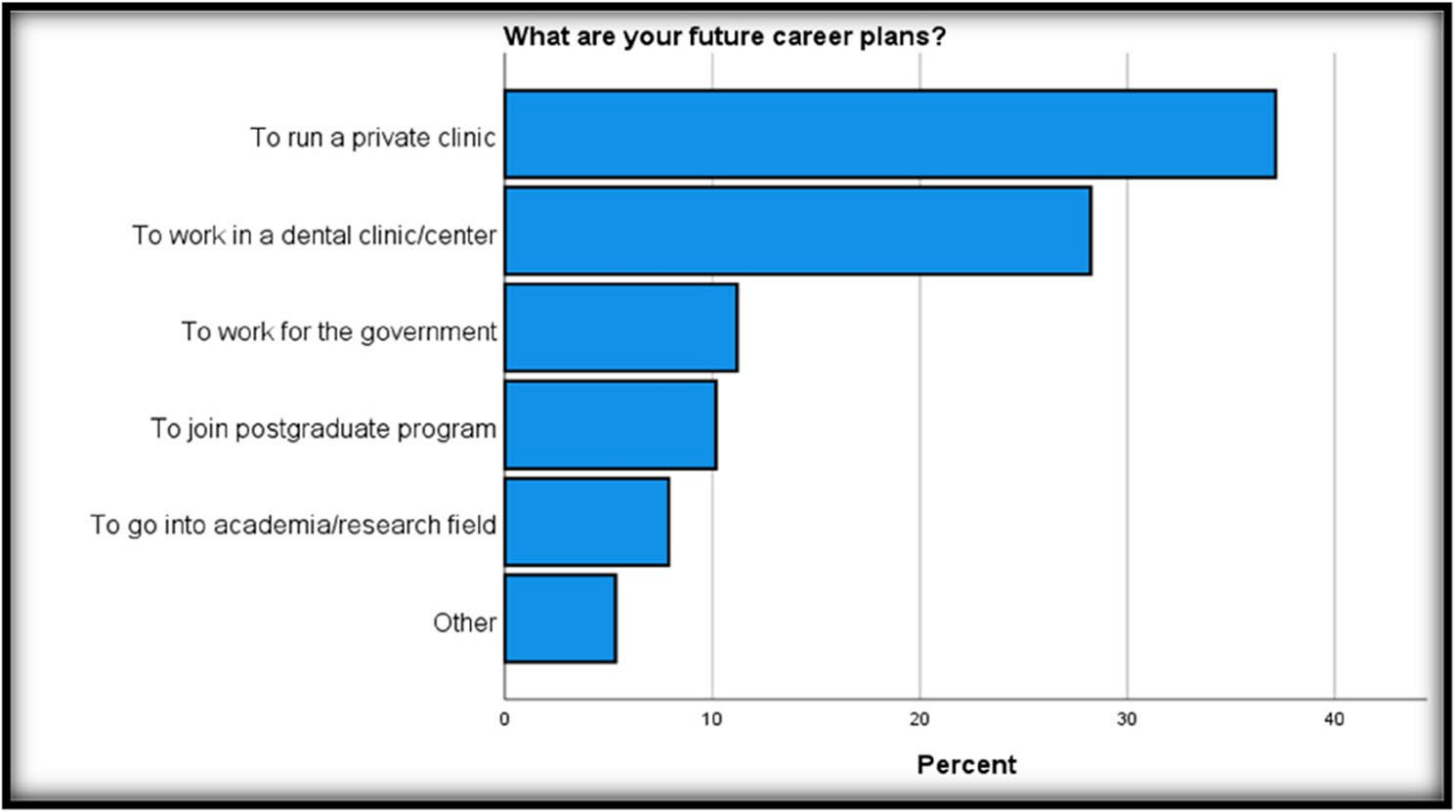
Demonstrates the overall future career plans of the respondents

A large proportion of students (68.2%) reported that they did not have a parent or close family member who is a dentist. The most frequently reported reason for choosing dentistry as a career was to be financially successful (32.8%), followed by chance (28.0%) and to be a highly respected member of the community (21.1%), where “chance” means by luck or assigning the student according to their score in high school. Running a private clinic was the most reported plan for future career (37.2%), followed by working in a dental clinic or center (28.2%), as shown in (Fig. 1). The type of university influenced the students’ choice for their future career path (*P* = 0.03). Government employment was more popular among government students, while working in a dental clinic or center was more favored by private students, as shown in (Table 1).

**Table 1:** Describes students’ future career plans in relation to the type of university

		Type of university				X^2*^	P value
		Governmental		Private			
		N	%	N	%		
What are your future career plans?	To run a private clinic	74	38.1%	72	36.2%	12.05	0.03 (s)
	To work in a dental clinic/center	49	25.3%	62	31.2%		
	To work for the government	30	15.5%	14	7.0%		
	To go into academia/research field	18	9.3%	13	6.5%		
	To join postgraduate program	16	8.2%	24	12.1%		
	Other	7	3.6%	14	7.0%		

*Chi-square test

- Key of significance: - *p* < 0.05 = statistically significant (s)

- *p* > 0.05 = statistically non-significant (ns)

**Fig. 2:**
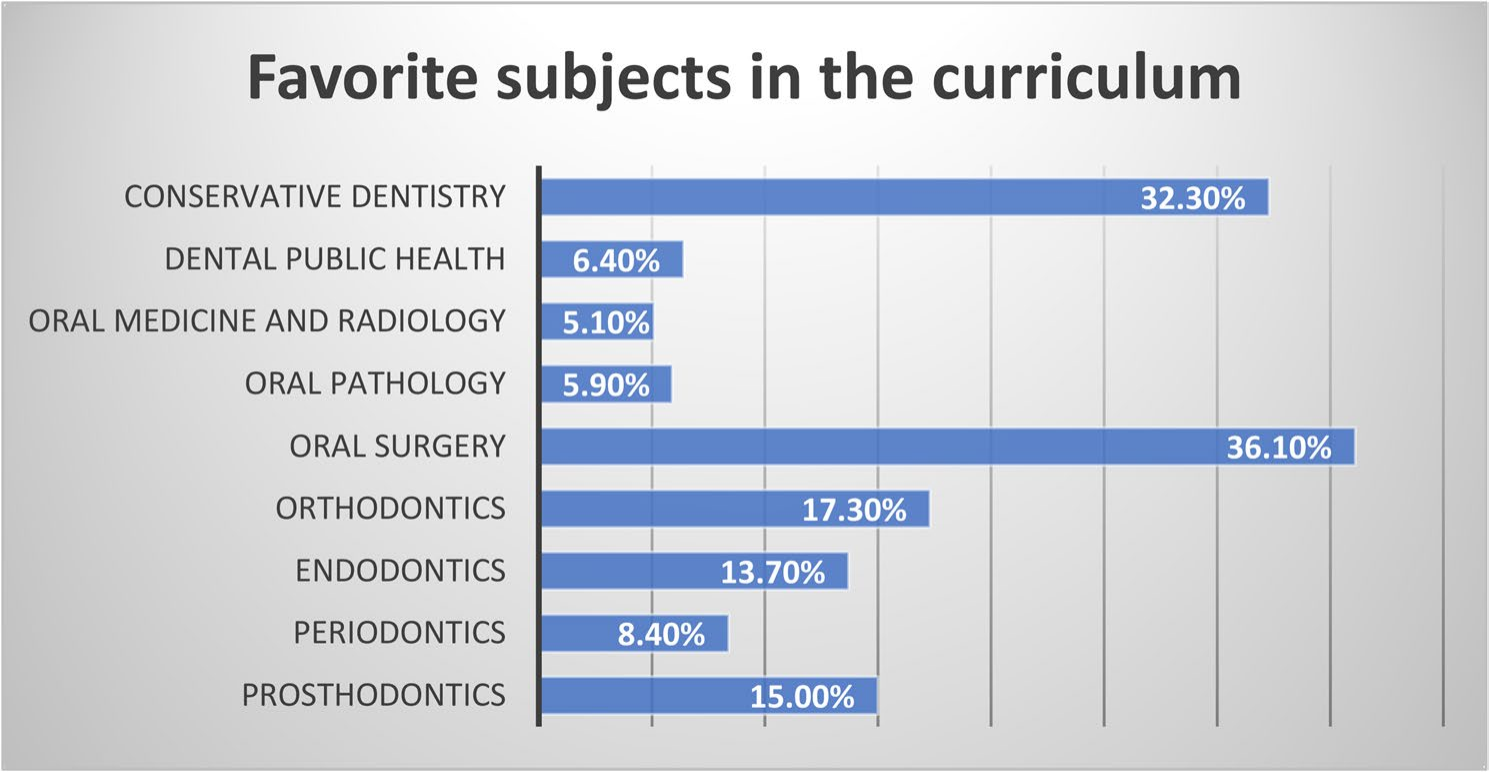
Demonstrates the distribution of students’ favorite subjects in the undergraduate dental curriculum among both governmental and private universities

Oral surgery and conservative dentistry were the most popular subjects among the students in their undergraduate curriculum, favored by 36.1% and 32.3% of students, respectively, followed by orthodontics (17.3%) and prosthodontics (15.0%), endodontics (13.7%) and periodontics (8.4%) while DPH was less popular, preferred by only 6.4% of students, followed by oral pathology (5.9%), and oral medicine and radiology (5.1%), as shown in (Fig. 2). Statistically significant differences between governmental and private universities in the choice of the favorite subject were only observed in endodontics and oral pathology.

Oral surgery (chosen by 31.6%) and conservative dentistry (chosen by 20.6%) were also the most favored specialties for postgraduate studies, while DPH was chosen by only 3.6% of the students.

About half (51.1%) of the students reported willingness to work in public service. A large proportion found the subject of DPH either interesting (30.0%) or useful (28.8%), while a few described it as boring (15.3%), difficult (10.4%), or useless (4.1%), with a statistically significant association between their opinions regarding DPH as a subject and their attitudes towards a career in the specialty (*P* < 0.001). Preventive dentistry was the most interesting field of DPH (58.3%), followed by oral health promotion (28.2%) and epidemiology (10.2%).

About half of the students (49.9%) reported a positive attitude towards a career in DPH, as shown in (Table 2). More than half of the students in both governmental (51.5%) and private universities (48.2%) expressed a positive attitude towards a career in DPH, with neutral and negative attitudes distributed similarly, as shown in (Table 3). The chi-square test showed no significant association (*p* = 0.41), indicating that the type of university does not influence students’ attitudes toward DPH.

**Table 2:** Shows the overall attitude score toward DPH

Total attitude score		Min.	Max.	Mean	SD
		0.00	52.00	33.51	7.59
		N		%	
Total attitude	Negative	15		3.8%	
	Neutral	182		46.3%	
	Positive	196		49.9%	
	Total	393		100.0%	

**Table 3:** Analyzes the relationship between the type of university and students’ attitudes

		Negative attitude (*N* = 15)		Neutral attitude (*N* = 182)		Positive attitude (*N* = 196)		X^2*^	P value
		N	%	N	%	N	%		
Type of university	Governmental	5	2.6%	89	45.9%	100	51.5%	1.77	0.41 (ns)
	Private	10	5.0%	93	46.7%	96	48.2%		

*Chi-square test

- Key of significance: - *p* < 0.05 = statistically significant (s) - *p* > 0.05 = statistically non-significant (ns)

Although only a small proportion of the students identified DPH as their career of interest (1.8% strongly agreed and 4.6% agreed), with the majority undecided (38.2%), disagreeing (42%) or strongly disagreeing (13.5%), many students acknowledged positive aspects of the field, such as its emotional rewards (64.9% agreeing or strongly agreeing), its role in raising oral health awareness (78.4%), its contribution to helping people (85.3%), and opportunities in research (71%), academics (63.4%), and working abroad (69.2%) as shown in (Table 4).

**Table 4:** Presents detailed attitudes of students toward considering DPH as a future career

	Strongly agree		Agree		Undecided		Disagree		Strongly disagree	
	*N*	%	*N*	%	*N*	%	*N*	%	*N*	%
It is my career of interest	7	1.8%	18	4.6%	150	38.2%	165	42.0%	53	13.5%
I am inspired by an eminent public health dentist/teacher	49	12.5%	149	37.9%	96	24.4%	68	17.3%	31	7.9%
The specialty is emotionally rewarding	73	18.6%	182	46.3%	85	21.6%	36	9.2%	17	4.3%
The specialty offers easy employment	48	12.2%	116	29.5%	127	32.3%	67	17.0%	35	8.9%
The specialty is a vital component in creating oral health awareness in the society	135	34.4%	173	44.0%	55	14.0%	18	4.6%	12	3.1%
The specialty offers better financial position than other specialties	60	15.3%	117	29.8%	125	31.8%	62	15.8%	29	7.4%
The specialty offers a challenging job	81	20.6%	159	40.5%	96	24.4%	39	9.9%	18	4.6%
The specialty offers a good chance to work in the academic field	82	20.9%	167	42.5%	93	23.7%	34	8.7%	17	4.3%
The specialty offers a good opportunity to work in the research field	102	26.0%	175	44.5%	74	18.8%	30	7.6%	12	3.1%
The specialty offers good opportunities to continue education/work abroad	104	26.5%	168	42.7%	84	21.4%	28	7.1%	9	2.3%
The specialty is more prestigious than other specialties	63	16.0%	119	30.3%	140	35.6%	49	12.5%	22	5.6%
It offers a chance to help people	185	47.1%	150	38.2%	35	8.9%	15	3.8%	8	2.0%
It helps in the development of personality in a society point of view	130	33.1%	197	50.1%	39	9.9%	16	4.1%	11	2.8%

Both male and female students showed nearly identical distribution across levels of attitude, meaning gender does not play a role in shaping students’ attitudes toward DPH (*P* = 0.97). The same observation was noticed among students from urban and rural areas, meaning area of residence does not play a role in shaping students’ attitudes toward DPH either (*P* = 0.4).

Most of the students (80%) who favored the subject of DPH in their undergraduate curriculum reported a positive attitude towards the specialty, while 20% of them reported a neutral attitude, demonstrating a statistically significant relationship (*P* = 0.01) between the choice of DPH and their attitude. However, none of the other subjects demonstrated such a relationship. Students’ choice of DPH for their postgraduate studies did not influence their attitude towards the specialty (*P* = 0.69).

A statistically significant relationship (*P* < 0.001) was observed between students’ opinions of the DPH subject and their attitudes toward pursuing it as a career (Table 5). Students who found the subject interesting were predominantly in the positive-attitude group (70.3%), and those who found it boring were mostly in the neutral-attitude group (56.7%).

**Table 5:** Shows how students with different overall attitude levels perceived the subject of DPH

		Negative attitude (*N* = 15)		Neutral attitude (*N* = 182)		Positive attitude (*N* = 196)		X^2*^	P value
		N	%	N	%	N	%		
How do you find the subject of dental public health right now?	Interesting	3	2.5%	32	27.1%	83	70.3%	43.38 FE	< 0.001 (s)
	Boring	5	8.3%	34	56.7%	21	35.0%		
	Easy	2	4.4%	24	53.3%	19	42.2%		
	Difficult	0	0.0%	26	63.4%	15	36.6%		
	Useful	2	1.8%	57	50.4%	54	47.8%		
	Useless	3	18.8%	9	56.3%	4	25.0%		

*Chi square test

- Key of significance: - *p* < 0.05 = statistically significant (s)

- *p* > 0.05 = statistically non-significant (ns)

The multivariable logistic regression analysis demonstrated that gender, type of university, and attitude level toward the specialty were statistically significant predictors, whereas age, residence, and year of study were not significantly associated with career intention, as shown in (Table 6).

**Table 6:** Demonstrates the result of the multivariable regression analysis

	B	S.E.	Sig.	Odds ratio	95% C.I. for odds ratio	
					Lower	Upper
Age (years)	− 0.011	0.011	0.303	0.989	0.967	1.010
Male gender	− 0.501	0.216	0.021	0.606	0.396	0.926
Urban residence	0.389	0.243	0.110	1.475	0.915	2.376
Private university	0.509	0.251	0.042	1.664	1.018	2.722
Year of study*						
Fifth year	− 0.470	0.399	0.238	0.625	0.286	1.365
Internship	0.565	0.454	0.213	1.759	0.723	4.278
Attitude**						
Average attitude	1.311	0.669	0.050	3.711	1.000	13.766
High attitude	2.044	0.671	0.002	7.723	2.071	28.793

*Reference group: 4th year **Reference group: low attitude

Gender was identified as a statistically significant predictor (*p* = 0.021; OR = 0.606; 95% CI: 0.396–0.926). The odds ratio less than one indicates that male students were significantly less likely to express intention to pursue a DPH career compared with female students, after controlling for other variables in the model.

The type of university also showed a statistically significant association with intention to pursue a DPH career (*p* = 0.042; OR = 1.664; 95% CI: 1.018–2.722). Students enrolled in private universities were approximately 1.7 times more likely to report intention to pursue a DPH career compared with students in governmental universities.

Attitude toward the specialty was the strongest predictor of intention to pursue a DPH career. Students with neutral attitude scores were approximately 3.7 times more likely to intend to pursue a DPH career compared with those having negative attitude scores (OR = 3.711; *p* = 0.050; 95% CI: 1.000–13.766), showing borderline statistical significance. Furthermore, students with positive attitude scores were about 7.7 times more likely to express intention to pursue a DPH career (OR = 7.723; *p* = 0.002; 95% CI: 2.071–28.793), indicating a strong and statistically significant association.

## Discussion

The importance of this study is that it explores the attitudes of dental students in Egypt towards DPH as a career choice in general, and specifically, compares the differences in their attitudes between governmental and private universities. By examining students’ demographic characteristics, academic preferences, career aspirations, and attitudes toward DPH, this discussion places the study’s findings in the context of emerging national and international trends and sheds light on patterns and factors that may be influencing workforce distribution in Egypt and explores aspects that may explain the inconsistencies between students’ overall positive attitudes toward DPH and their refrainment from pursuing it professionally, while comparing these results with international studies. The findings of this study support the null hypothesis since no significant association (*p* = 0.41) was observed between the type of university and the attitudes of the students.

Findings emphasize the need to shift the scope and effort of the health sector from treatment to prevention of diseases. Therefore, the demand for community health workers in various sectors and fields of medicine, including dentistry, is increasing. It is important to attract more dental students to DPH and community-oriented careers to meet this increasing demand. Findings also highlight the importance of expanding the breadth of this specialty and enhancing its practical application by adopting evidence-based strategies and integrating practical learning into institutional structures.

The nearly balanced number of males and females, which is in accordance with the study conducted by Mekhemar et al. [7]. means that the sample was equally distributed among both genders, which strengthens the results of this study. The majority of students lived in urban areas and might be less aware of rural oral health issues and the vital role of public health, and have less exposure to rural populations, which, in Egypt, are much more likely to be underserved or marginalized [31]. Most students did not have a dentist in their close family and thus have less exposure to private practice norms and may be more open to DPH or community-oriented careers, while those students with dentists in their family may be inclined to follow family traditions, running an already existing dental family business [32]. The most frequently reported reason for choosing dentistry as a profession was financial success, which might be attributed to the fact that dentistry is often viewed as a profession with strong economic prospects and high income [33]. In contrast, Dutta et al. [20] found that the majority of students reported joining the profession by chance, mainly due to their interest in medicine, and their inability to secure a medical seat.

Logically, running a private clinic and working in a dental clinic or center were the most common goals for a future career, since both responses are the most highly paid positions for a dentist in Egypt, compared to the low salary of Egyptian dentists working only in government postings [34]. In contrast, Sharma et al., [30] Dutta et al. [20] and Devi et al. [23] reported that most students wanted to pursue postgraduate specialist training.

Among the undergraduate subjects, oral surgery was the most popular, followed by conservative dentistry, orthodontics, and prosthodontics, while DPH, which is a mandatory subject of the curriculum of all Egyptian dental schools, [5] was only favored by 6.4% of students. Dutta et al. [20] reported a similar observation where DPH was only favored by 1% of students. A similar, but not identical, pattern was observed when the students were asked about their department of choice for postgraduate studies. Oral surgery remained the most popular, followed by conservative dentistry, orthodontics, and endodontics, while DPH was the second least popular choice. Conservative dentistry was the most popular choice reported by Sharma et al. [30] and Dutta et al. [20], who also reported that DPH was the choice of only 7.7% of the students. In line with our study, the top three specialties in the study conducted by Devi et al. [23] were oral surgery, orthodontics, and conservative dentistry. These findings are logically consistent, since those clinical specialties are the most highly paid among the fields of dentistry [35].

About half of the students showed interest in working in public service for their community. Sharma et al. [30] Shaktawat et al. [21] Dutta et al. [20] and Devi et al. [23] reported a higher proportion among the students. This disparity could be attributed to the vast economic discrepancy between governmental salaries and the high potential earnings of the private sector [34]. Other reasons could be the predominantly urban background of the participants that might influence their hesitation to engage in public service, and the lack of a distinct, high-status professional identity for DPH in Egypt, where it is often integrated into other departments, [5] which may lead students to view public health jobs as secondary or temporary roles rather than prestigious career prospects.

Almost half of the students had positive attitudes towards pursuing a career in DPH, while only a small proportion had negative attitudes. The students agreed that the specialty is emotionally rewarding, has a vital role in raising oral health awareness, offers a chance to help people, and offers a good opportunity to work abroad, in the research field, and in the academic field.

Shaktawat et al. [21] and Dutta et al. [20] reported a higher proportion of students (32% and 16.5%, respectively) showing interest in DPH as a career. Naidu et al. [29] reported different proportions of students with high (35.4%), average (58.02%), and low (6.48%) levels of attitude, while Sharma et al. [30] reported high (33.52%), average (58.23%), and low (8.23%). Devi et al. [23] found a positive attitude among 68.4% of their sample. These findings could mean that the students are aware of the importance of the field of DPH, the vital role it plays in improving the oral health of the community, and the good opportunities it offers. However, in line with our findings, a small proportion of them considered a career in DPH and favored the subject of DPH in their undergraduate curriculum, and even fewer students were willing to continue to study the subject post-graduation. The students’ refrainment from pursuing a career in DPH could mean that they are unable to overcome the barriers imposed by the social norms of the dental community, where clinical practice and financial gain are often viewed as the most important aspects of a successful career [36–38]. 

The type of university did not influence students’ attitudes toward DPH as a career choice. This could be attributed to similar teaching environments adopted by both types of universities, a unified national curriculum, comparable clinical training, and standardization of educational quality. In contrast, Devi et al. [23] reported a significant difference in students’ attitudes between one governmental university and one private university in their study conducted in Puducherry, India.

Gender did not seem to influence the attitudes of students towards DPH in the present study. This finding reflects increasing gender equity and similar career aspirations. In contrast, Naidu et al. [29] reported that many more female students expressed a positive attitude compared to male students. Sharma et al. [30] also reported a statistically significant difference in attitude between both genders, while Devi et al. [23] in accordance with our findings, reported no significant difference between the two groups.

Regarding the students’ favorite subject in their undergraduate curriculum, a statistically significant relationship between the students’ choice and their attitude towards DPH was only observed with the students who preferred the subject of DPH (*P* = 0.01), where 4/5 of them expressed a positive attitude, while none of them expressed a negative attitude. This finding suggests that positive learning experiences and interest in DPH in undergraduate years play an important role in improving the students’ attitude towards the field and can motivate them to choose it as a future career. 

A statistically significant relationship between students’ opinions of the subject of DPH and their attitudes toward pursuing it as a career indicates that positive engagement with course content can lead to a positive attitude towards the specialty as a career path, and that lack of interest may reduce enthusiasm for pursuing DPH professionally, though it does not necessarily produce strong negative attitudes.

The multivariable logistic regression analysis for factors associated with intention to pursue a career in DPH further supports the findings of our study, regarding the distinction between the overall positive attitude of the students towards DPH and their willingness to pursue a career in the specialty. However, the finding that private university students demonstrated higher odds of pursuing a DPH career contradicts our previously stated assumption that governmental university students show more interest in the public sector. This may be explained by the resource-intensive educational environments in private institutions, which might foster a broader understanding of international health policy and research pathways.

The main strengths of the present study are its conduct at three different regions of Egypt, Greater Cairo, Nile Delta, and Upper Egypt, which strengthens the results of this study as this includes dental students from different social and cultural backgrounds in Egypt, and the inclusion of both governmental and private universities in the sample, comparing the attitudes, demographics, opinions and aspirations of the students between both types of universities.

Limitations of this study include its cross-sectional design, which did not allow us to know or predict whether the students’ opinions and attitudes towards DPH or their career intentions changed over time. Because only six universities were included out of more than forty dental schools that exist in Egypt, and since they were not randomly selected, the study results may not be generalizable to all dental students in the country. The sample size was not adjusted for a design effect, which may slightly reduce the study’s statistical power. The study was quantitative in nature and didn’t explore the exact reasons behind the students’ failure to choose DPH as their favorite subject or to consider it for their post-graduate studies. However, exploring the students’ attitudes further by qualitative surveys could be the basis for future research.

## Conclusions

The type of university, whether governmental or private, did not influence the students’ attitudes towards DPH as a career choice. Despite the students’ overall positive attitude towards DPH, few of them considered pursuing a career in the specialty.

Findings suggest the need to shift the scope and effort of the health sector from treatment to prevention of diseases, in developing countries, including Egypt, which significantly increases the demand for community health workers and underscores the importance of attracting dental students toward community-oriented career paths. To support this shift, DPH education in Egyptian dental schools must be reformed, moving away from traditional, lecture-based theoretical curriculum [5] toward one relying on clinical applications and fieldwork, which is essential to fostering the positive perceptions necessary for career recruitment. Incentivizing public health services, including DPH, and demonstrating their real-world impact and diverse future prospects, is essential to attract more students. Enhancing the visibility and professional prestige of DPH is essential, which can be achieved by projecting successful and inspiring DPH role models to serve as mentors for the next generation of dental professionals.

## Supplementary Information

Supplementary Material 1.

## Data Availability

All the data generated for this study are derived from participants’ responses to paper-based, hand-filled questionnaires, which were entered into a *Google Sheets* file. The data cannot be shared openly for privacy reasons.
