# Supplementary figures and images for: Attitudes of dental students in governmental versus private universities in Egypt towards acquiring a career in dental public health: a cross-sectional study
Source: BMC Med Educ. 2026 May 4;26:716. doi: 10.1186/s12909-026-09280-x (PMC13137723; doi:10.1186/s12909-026-09280-x)

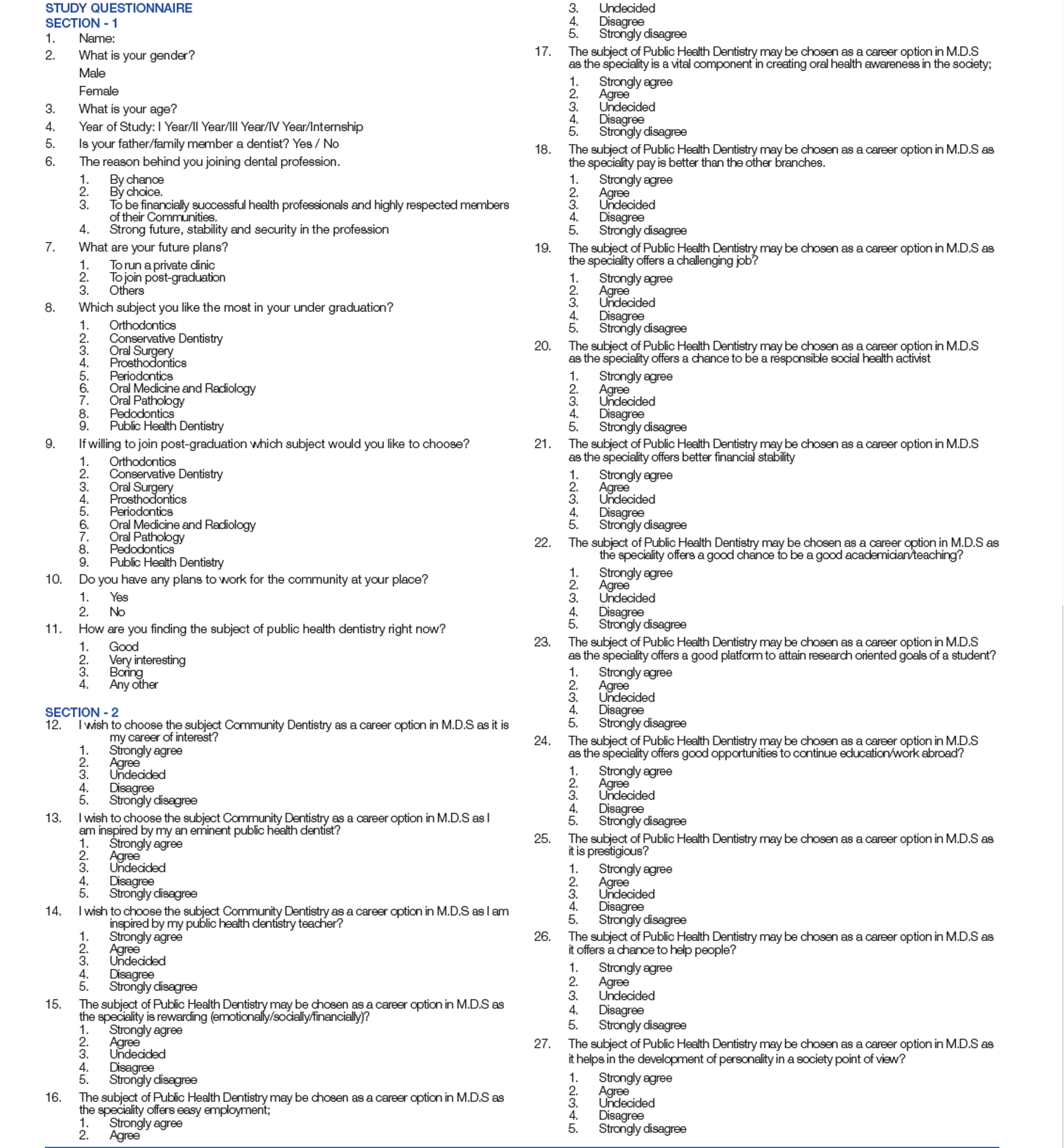

Supplement: Supplementary file 1 — Supplementary Material 1. [file 12909_2026_9280_MOESM1_ESM.png]
